# Next-Generation Sequencing from Bulked-Segregant Analysis Accelerates the Simultaneous Identification of Two Qualitative Genes in Soybean

**DOI:** 10.3389/fpls.2017.00919

**Published:** 2017-05-31

**Authors:** Jian Song, Zhen Li, Zhangxiong Liu, Yong Guo, Li-Juan Qiu

**Affiliations:** The National Key Facility for Crop Gene Resources and Genetic Improvement/MOA Key Labs of Crop Germplasm and Soybean Biology, Institute of Crop Science, Chinese Academy of Agricultural SciencesBeijing, China

**Keywords:** mapping by sequencing, next-generation sequencing, BSA, SNP-index, cotyledon color

## Abstract

Next-generation sequencing (NGS)-based bulked-segregant analysis (BSA) approaches have been proven successful for rapidly mapping genes in plant species. However, most such methods are based on mutants and usually only one gene controlling the mutant phenotype is identified. In this study, NGS-based BSA was employed to map simultaneously two qualitative genes controlling cotyledon color of seed in soybean. Yellow-cotyledon (YC) and green-cotyledon (GC) bulks from progenies of a biparental population (Zhonghuang 30 × Jiyu 102) were sequenced. The SNP-index of each SNP locus in YC and GC bulks was calculated and two genomic regions on chromosomes 1 and 11 harboring, respectively, loci *qCC1* and *qCC2* were identified by Δ(SNP-index) analysis. These two BSA-seq-derived loci were further validated with SSR markers and fine-mapped. *qCC1* was mapped to a 30.7-kb region containing four annotated genes and *qCC2* was mapped to a 67.7-kb region with nine genes. These two regions contained, respectively, genes *D1* and *D2*, which had previously been identified by homology-based cloning as being associated with cotyledon color. Sequence analysis of the NGS data also identified a frameshift deletion in the coding region of *D1*. These results suggested that BSA-seq could accelerate the mapping of loci controlling qualitative traits, even if a trait is controlled by more than one locus.

## Introduction

Identifying a gene or locus conditioning a trait is one of the major tools for characterization of gene function and eventually for the improvement of agronomic traits in crops (Takeda and Matsuoka, 2008). Conventional positional cloning and quantitative trait locus (QTL) mapping are powerful approaches for investigating the genetic control of phenotypic variation in agronomic traits (Burke et al., 2007). The initial stages of these gene-mapping approaches include genome-wide investigation of polymorphic molecular markers coupled with subsequent identification of the most promising candidate regions. Further steps involve fine mapping by increasing marker density across the target region and development of physical maps, followed by candidate gene isolation and validation (Peters et al., 2003; Gallavotti and Whipple, 2015). This strategy has been successfully used to identify several genes and QTLs with important effects in crops (Xia et al., 2012; Funatsuki et al., 2014). However, classical map-based gene cloning approaches are usually low-throughput and time-consuming.

Bulked segregant analysis (BSA) provides a simple approach for rapidly identifying molecular markers tightly linked to the causal gene underlying a given phenotype (Giovannoni et al., 1991; Michelmore et al., 1991). Starting with construction of a segregating population, two bulked DNA samples are generated from progenies with contrasting phenotypes and genotyped with molecular markers polymorphic between the parental lines (Quarrie et al., 1999). BSA technologies have been used in many organisms to map important genes (Mansur et al., 1993; Yi et al., 2006; Watanabe et al., 2011; Whipple et al., 2011). With the development of DNA sequencing technology, next-generation sequencing (NGS)-based BSA approaches dramatically accelerate the process of identifying causal genes (Schneeberger and Weigel, 2011).

Initially, NGS coupled with BSA in flowering plants was applied to identify causal genes for growth habit and leaf color in *Arabidopsis* (Schneeberger et al., 2009). Subsequently, many methods and pipelines have been developed in model plants *Arabidopsis* and rice (Austin et al., 2011; Uchida et al., 2011; Abe et al., 2012; Hartwig et al., 2012; Lindner et al., 2012; Fekih et al., 2013; Takagi et al., 2013b). These approaches have been successfully used to identify candidate genes for important traits or phenotypes in rice (Takagi et al., 2015; Zheng et al., 2016), maize (Liu et al., 2012; Haase et al., 2015), barley (Mascher et al., 2014), and soybean (Campbell et al., 2016; Dobbels et al., 2017). However, most of these map-by-sequencing methods are based on mutants and usually only one gene controlling the mutant phenotype is identified. Few studies were able to map more than one gene simultaneously using populations derived from crop germplasm.

Soybean is one of the most widely planted legume crops all over the world. Its seed contains about 20% oil and 40% protein and constitutes an important source of vegetable oil and plant protein for human and animal consumption. In addition, soybean components such as α-linolenic acid and isoflavones have beneficial effects on human health. The soybean genome is about 1.1 Gb, 40–60% of which is repetitive sequence (Schmutz et al., 2010). Owing to the low genetic variation, large and complex genome and low efficiency of genetic transformation, gene identification and isolation in soybean lag behind corresponding activities in other crops. Only a few genes controlling traits including stem growth habit, seed number per pod, hard-seededness, and salt tolerance have been identified by positional cloning approach (Jeong et al., 2012; Guan et al., 2014; Ping et al., 2014; Sun et al., 2015). Developing methods for rapidly mapping genes controlling important agronomic traits is important for the functional study of soybean genes.

Cotyledon color in the mature seed is an important morphological trait for soybean breeding and germplasm classification. Most soybean cultivars have yellow cotyledons and only a few have green ones. Three inheritance patterns (maternal inheritance and double- and single-gene inheritance) for soybean cotyledon color have been identified and classical genetics methods have revealed that several loci including *D1, D2*, and *cytG* regulate this trait (Woodworth, 1921; Guiamet et al., 1991). Recently, *D1* and *D2* were cloned as homologs of *STAY-GREEN* (*SGR*) genes by the homology-based cloning method (Fang et al., 2014; Nakano et al., 2014).

In the present study, a genome-wide NGS-based BSA mapping approach was implemented in a soybean biparental population for cotyledon color controlled by two genes. After progenies derived from the crossing of two soybean accessions with distinct cotyledon colors were phenotyped, yellow-cotyledon (YC) and green-cotyledon (GC) bulks were constructed and sequenced along with their parental lines. Associated regions were identified using the Δ(SNP-index) method after SNPs among parental lines and DNA bulks were called. Two associated loci were validated and fine-mapped to 30.7 and 67.7-kb intervals by marker-based classical gene mapping. Two previously identified stay-green genes were located in fine-mapped regions and a sequence variant of the *D1* gene was identified by analysis of whole genome sequencing data, indicating that BSA combined with high-throughput sequencing can be used for rapid mapping of qualitative traits, even if a trait is controlled by more than one locus.

## Materials and Methods

### Plant Materials

*Glycine max* cv. Zhonghuang 30 (ZH30), with yellow cotyledons, and Jiyu 102 (JY102), with green cotyledons, were obtained from the National Soybean Genebank, Institute of Crop Science, Chinese Academy of Agricultural Sciences. ZH30 and JY102 were crossed and confirmed F_1_ plants were self-fertilized to develop segregating populations. The cotyledon colors of all F_1_ and F_2_ seeds and selected F_2:3_ populations were recorded. The chi-square (χ^2^) test was used to evaluate the fit of observed to expected segregation ratios in all populations.

### Construction of Sequencing Libraries and Illumina Sequencing

Genomic DNA was isolated from young leaves of soybean using a genomic DNA purification kit (Thermo Fisher Scientific Inc., United States) according to the manufacturer’s protocol. DNA samples were quantified using a Quawell Q5000 spectrophotometer (Quawell Technology, Inc., United States). Two bulks were generated by pooling equal amounts of DNA from 30 lines with green cotyledons and 30 with yellow cotyledons. About 5 μg of DNA from two bulks and two parental lines were used to construct paired-end sequencing libraries, which were sequenced on an Illumina HiSeq^TM^ 2500 platform.

After removing adapter and low quality reads, the clean reads were further rechecked for quality using FASTQC^1^. High-quality sequences were aligned and mapped to the *Glycine max* Wm82.a2.v1 reference genome from Phytozome^2^ using BWA with default parameters (Langmead and Salzberg, 2012). GATK (Genome Analysis Toolkit) was used to call SNPs and small indels across parental lines and bulks (McKenna et al., 2010).

### SNP-Index Analysis

Homozygous SNPs between parental lines and high-quality SNPs (minimum sequence read depth: 10 with SNP base quality ≥ 100 in bulks) were selected for SNP-index analysis. A SNP-index was calculated at each SNP position for both the YC and GC bulks using the base in JY102 as alternative base (Abe et al., 2012; Takagi et al., 2013b). Thus, the SNP-index was assigned as 0 or 1, when entire short sequence reads contained genomic fragments derived from ZH30 or JY102, respectively. A Δ(SNP-index) was calculated by subtraction of the YC index from the GC index (Fekih et al., 2013; Takagi et al., 2013a; Das et al., 2015; Singh et al., 2015). Thus, a high Δ(SNP-index) value of a SNP locus is indicative of an allele that was both very frequent in the GC bulk and depleted in the YC bulk. A *P*-value for Fisher’s exact test performed between the GC and YC bulks at each SNP locus was also calculated.

### Sliding-Window Analysis

The average distributions of the SNP-index and Δ(SNP-index) were estimated in a given genomic interval by a sliding window approach with a 2-Mb window size and 10-kb step, and were plotted to generate SNP-index plots for all soybean chromosomes. Regions in which the average Δ(SNP-index) of a locus was significant larger than surrounding region and windows showed an average *P*-value < 0.05 were considered candidate genomic regions harboring a locus associated with cotyledon color.

### Validation and Fine Mapping of Candidate Regions

BARCSOYSSR and SNP markers polymorphic between the two parental lines were developed in the predicted candidate regions of *qCC1* and *qCC2* (Song et al., 2010; **Supplementary Table S1**). One hundred lines with cotyledon color segregation fitting a 3:1 ratio were selected for genotyping. These lines were classified into two groups after genotyping *qCC1* and *qCC2* loci, with cotyledon color of the members in one group only controlled by *qCC1* and in the other only controlled by *qCC2*. Based on the difference between the genotypes as assessed using polymorphic markers, recombinants were identified in F_2:3_ populations and used to refine the locations of *qCC1* and *qCC2*.

### RNA Extraction, cDNA Synthesis, and PCR Amplification

Total RNA was isolated from soybean leaves using TRIzol reagent (Invitrogen, United States) according to the manufacturer’s protocol. The quality and quantity of RNA samples were assessed on a Quawell Q5000 spectrophotometer (Quawell Technology, Inc., United States). cDNA was synthesized using the PrimerScrip^TM^ RT Reagent Kit (Takara, Japan) after removal of genomic DNA from the RNA. Gene-specific primers (**Supplementary Table S1**) were used to amplify the promoter and genomic sequences of *Glyma.01g214600* and the coding sequence of *Glyma.11g027400*. PCR products were analyzed on 1% agarose gels by electrophoresis, followed by sequencing and alignment. The quantitative RT-PCR was carried out on an Applied Biosystems 7300 Real-Time PCR System, using SYBR Premix Ex Taq kit (TaKaRa, Japan). The relative expression level of each gene was calculated using 2^-ΔΔt^ method (Livak and Schmittgen, 2001).

### Data Availability

Whole-genome sequencing data for ZH30, JY102, and the two bulks are available in the NCBI Sequence Read Archive under accession numbers SRX2789685(ZH30), SRX2789686(JY102), SRX2789687(YC bulk), and SRX2789688(GC bulk).

## Results

### Genetic Analysis of the Segregating Population

**FIGURE 1:**
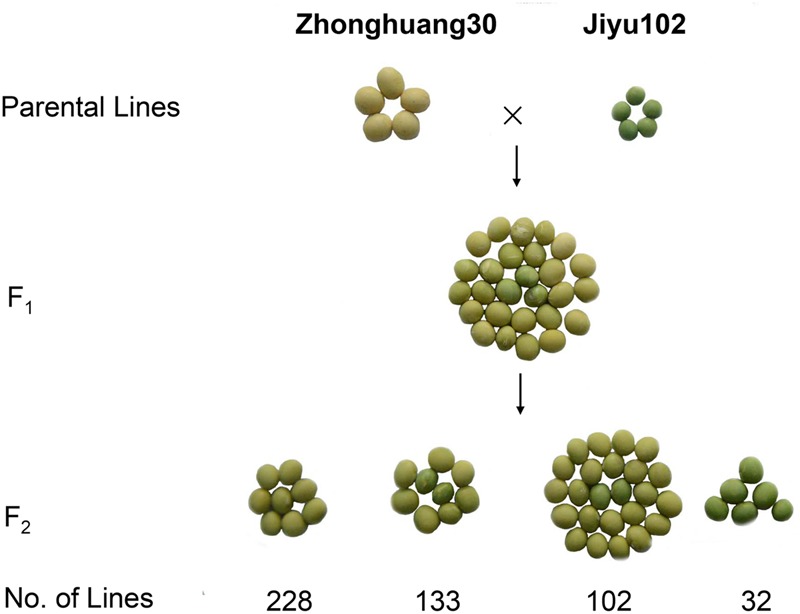
Genetic analysis of cotyledon color in a cross of ZH30 and JY102.

To investigate the inheritance of cotyledon color in soybean, two soybean cultivars, Zhonghuang 30 (ZH30) with yellow cotyledon and Jiyu 102 (JY102) with green cotyledon were used to develop segregating populations. All the hybrid seeds with ZH30 as maternal donor showed the YC phenotype and the seeds of F_1_ plants segregated for cotyledon color. Detailed analysis revealed that the segregation of cotyledon color in seeds of F_1_ plants all fit a 15:1 (yellow:green) segregation ratio with χ^2^ value ranging from 0.0008 to 1.79, all well below 3.84, the critical value for a significant difference (**Table 1**). Moreover, a segregating F_2_ population (*n* = 495) was developed and phenotypic evaluation showed that F_2:3_ lines derived from all 32 plants with green cotyledon in the F_2_ generation showed green cotyledons. Lines with yellow cotyledon in the F_2_ produced 228 F_2:3_ lines with yellow cotyledon and 133 and 102 F_2:3_ lines segregating in ratios of about 3:1 and 15:1, respectively (**Figure 1**). All of these results suggested that cotyledon color in this cross was controlled by two genes and that the green cotyledon trait carried by JY102 was recessive.

**Table 1:** Phenotype of seed cotyledon color evaluated in 11 F_2_ populations derived from a cross between ZH30 and JY102.

Populations	Total number of seeds	Seeds with yellow cotyledon	Seeds with green cotyledon	Observed ratio	χ^2^ (15:1)	*P*-value
130028-1	314	295	19	15.5:1	0.0008	0.8841503
130028-3	341	319	22	14.5:1	0.0018	0.8777637
130028-4	247	234	13	18.0:1	0.2594	0.5217025
130029-1	270	251	19	13.2:1	0.1669	0.5931627
130029-2	252	232	20	11.6:1	0.9524	0.2687178
130030-1	248	234	14	16.7:1	0.0688	0.6939535
130030-2	374	347	27	12.9:1	0.4456	0.4387141
130030-3	247	231	16	14.4:1	0.0003	0.8824539
130030-4	258	244	14	17.4:1	0.1747	0.5846935
130034-1	337	316	21	15.0:1	0.0097	0.9887781
130034-2	302	277	25	11.1:1	1.7881	0.1453782

### Construction and Sequencing of BSA Pools

Based on the phenotypic investigation, DNA from 30 individuals with yellow or green cotyledon each was pooled separately into a YC bulk and a GC bulk. DNA of each parental line isolated from leaves of 10 plants was also prepared for sequencing. These four DNA samples were used to construct libraries and subjected to whole-genome sequencing on the Illumina HiSeq^TM^ 2500 platform. After filtering, 144.6 Gb of clean data were obtained with average Q20 of 91.8% and Q30 of 85.2% (**Table 2**), indicating the high quality of the sequencing data.

**Table 2:** Summary of Illumina sequencing data.

Sample ID	YC-bulk	GC-bulk	ZH30	JY102
Clean reads	486,749,106	467,745,622	108,602,086	84,534,592
Clean bases	61,327,351,325	58,897,998,134	13,683,105,387	10,650,695,091
Q20 (%)	91.6	91.4	91.5	92.6
Q30 (%)	85.1	85.0	85.1	85.7
Mapped ratio (%)	94.7	93.8	94.8	94.6
Average depth	59×	53×	12×	9×
Coverage_ratio_1× (%)	95.2	93.5	93.2	89.7
Coverage_ratio_5× (%)	90.9	85.0	76.8	69.2
Coverage_ratio_10× (%)	87.9	80.0	57.4	44.1

Alignment with the Williams 82 reference genome allowed 93.8–94.8% of the clean reads to be mapped. The average sequencing depths for DNA bulks were 53–59 and those of the parental lines were 9–12. More than 89.7% of the genome had at least 1× coverage in all four samples and at least 80.0% had at least 10× coverage in the bulks. SNPs and small indels were first called between parental lines and the Williams 82 reference genome and then putative variations between parental lines were identified by selection SNPs or small indels that were unique to a single parent. Finally, a total of 1,084,921 SNPs and 157,839 small indels were identified between parental lines ZH30 and JY102.

### BSA Mapping Using the Sequencing Data

**FIGURE 2:**
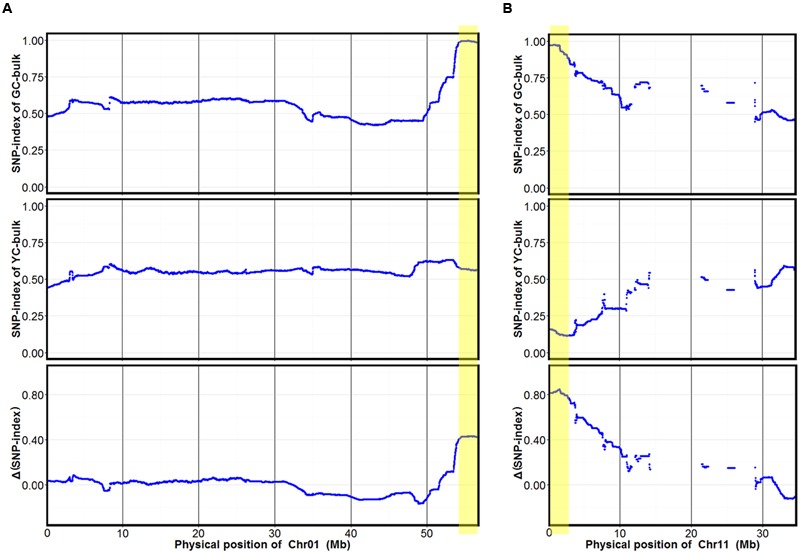
SNP-index of GC and YC bulks and Δ(SNP-index) plots generated by sliding-window analysis on soybean chromosomes 1 **(A)** and 11 **(B)**. The *X*-axis shows physical positions of chromosomes and the *Y*-axis the average SNP-index in each 2-Mb physical interval with a 10-kb sliding window. Two candidate genomic regions (marked in yellow) were defined using the criteria of average SNP-index > 0.9 in the GC bulk and average *P*-value < 0.05.

To identify markers associated with cotyledon color, SNP-index of each SNP locus in YC and GC bulks was calculated using high-quality SNPs, those with quality score ≥ 100 and read depth ≥ 10. The average SNP-index in YC and GC bulks and Δ(SNP-index) between the GC and YC bulks across a 2-Mb genomic interval were measured using a 10-kb sliding window and plotted for all 20 chromosomes of the soybean genome (**Supplementary Figure S1**). Fisher’s exact test was also performed for the YC and GC bulks at each SNP locus and the average *P*-values for SNPs located in each sliding window were calculated. Although many peaks were identified in SNP-index plotting of the YC and GC bulks, only two major peaks with statistical significance were identified in Δ(SNP-index) association analysis and were assigned as the candidate regions of the two genes controlling cotyledon color in this population (**Figure 2** and **Supplementary Figure S1**). These two candidate regions (designed as *qCC1* and *qCC2*) were located in the intervals 54.15–56.83 Mb on chromosome 1 and 0–2.68 Mb on chromosome 11, displaying an average SNP-index > 0.9 in the GC bulk and an average *P*-value < 0.05 (**Figure 2**). For the candidate region of *qCC1*, 2,843 SNPs between parental lines were identified and 2,284 of them had a SNP-index of 1.0 in the GC bulk (indicating that the entire short sequence reads contained genomic fragments derived from JY102). Of all these SNPs, 251 could result in changes in coding sequences (**Supplementary Table S2**). The candidate region of *qCC2* contained 1,237 high-quality SNPs between parental lines, of which 870 SNPs had a SNP-index of 1.0 in the GC bulk and 102 could result in changes in coding sequences (**Supplementary Table S3**).

### Validation of the BSA Mapping Results

**FIGURE 3:**
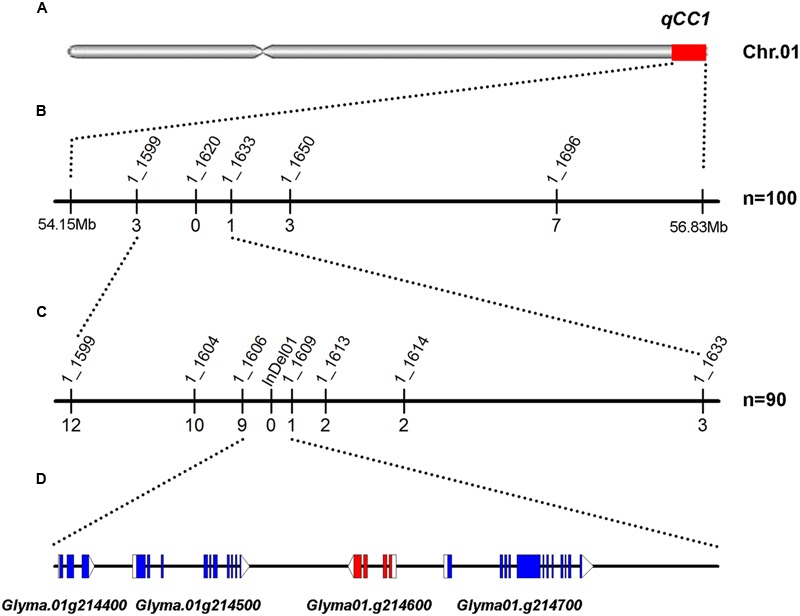
Mapping and fine mapping of the *qCC1* locus. **(A)** Chromosomal location of *qCC1* identified by NGS-based BSA on chromosome 1. **(B)** Coarse mapping of the *qCC1* locus. Vertical lines indicate polymorphic SSR markers. Names of markers are shown above the line and the number of recombinants between *qCC1* and each marker is shown below the line. **(C)** Fine mapping of *qCC1* with genotyping data from newly developed polymorphic markers. **(D)** Candidate genes in the fine-mapping region. Blue/purple rectangles, blank rectangles, and blank triangles represent exons, 5′ UTRs, and 3′ UTRs, respectively.

**FIGURE 4:**
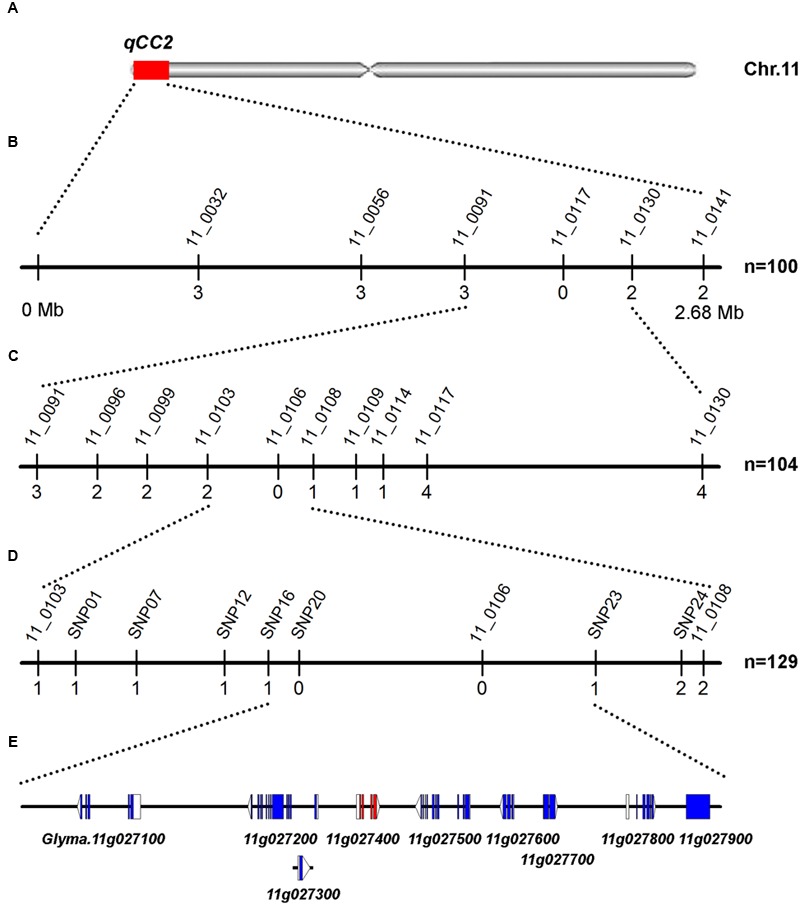
Mapping and fine mapping of the *qCC2* locus. **(A)** Chromosomal location of *qCC2* identified by NGS-based BSA method on chromosome 11. **(B)** Coarse mapping of *qCC2* locus. Vertical lines indicate polymorphic markers. Names of markers are shown above the line and the number of recombinants between *qCC2* and each marker is shown below the line. **(C,D)** Fine mapping of *qCC2* with genotyping data from newly developed polymorphic markers. **(E)** Candidate genes in the fine-mapping region. Blue/purple rectangles, blank rectangles, and blank triangles represent exons, 5′ UTRs, and 3′ UTRs, respectively.

To validate the candidate regions identified by BSA mapping, 11 SSR markers polymorphic between two parental lines in candidate regions were used for identifying genotypes in the F_2_ segregating population. One hundred lines with seed cotyledon color segregating in a 3:1 ratio were selected for genotyping in order to avoid the influence of the other locus. A total of 200 DNA samples were isolated and genotyping results showed that cotyledon colors in 52 of these 100 lines were regulated by *qCC1* in the recessive *qcc2* background and those in 48 lines by *qCC2* under the recessive *qcc1* background. Identification of the recombinants in these lines revealed that *qCC1* was located between markers BARCSOYSSR_01_1599 and 01_1633 in a 395-kb region and that *qCC2* was located in a 684-kb region between markers BARCSOYSSR_11_0091 and 11_0130 (**Figures 3, 4**), validating the accuracy of the NGS mapping result.

### Fine Mapping of *qCC1* and *qCC2* by Polymorphic Marker Development

To further delineate the *qCC1* locus, 70 randomly selected seeds with green cotyledons and 20 seeds with yellow cotyledons were used for identification of recombinants between markers BARCSOYSSR_01_1599 and 01_1633. The result showed that 19 plants had recombinant exchanges at either end of the *qCC1* region. Six polymorphic markers were then developed between these two markers and subsequent marker–phenotype analysis allowed progressive refinement of the *qCC1* region into a 30.7-kb region between markers BARCSOYSSR_01_1606 and 01_1609 (**Figure 3**). Four genes were annotated in the candidate region of *qCC1* according to the Wm82.a2.v1 gene set of the soybean reference genome. The candidate region contained 39 SNPs, of which three were located in exons (two synonymous and one non-synonymous variant) of two genes and 18 in introns or UTRs of three genes. The candidate region contained 15 small indels of size 1–10 bp, with two of them located in genes (including an exon of *Glyma.01g214600* and an intron of *Glyma.01g214700*) (**Supplementary Table S4**).

For the *qCC2* locus, 100 randomly selected seeds with green and four seeds with yellow cotyledons were used for identification of recombinants using markers BARCSOYSSR_11_0091 and 11_0130. The identified recombinants were genotyped with eight polymorphic SSR markers and *qCC2* was mapped to the interval between BARCSOYSSR_11_0103 and 11_0108. Another three recombinants were identified from other lines and seven SNP markers were used for fine-mapping the *qCC2* locus. Finally, *qCC2* was mapped between SNP16 and SNP23 in a physical interval of 67.7 kb (**Figure 4**). This region contained nine annotated genes, in which 15 SNPs and one small indel were identified between parental lines. None of these SNPs altered amino acid sequences of encoded proteins. Although a small indel resulted in a frameshift of the *Glyma.11G027800* gene, this gene may not be the candidate gene because this alteration occurs in the non-mutated line ZH30 but not the mutated line JY102 (**Supplementary Table S5**).

### Validation of the Causal Genes in Fine-Mapping Regions

**FIGURE 5:**
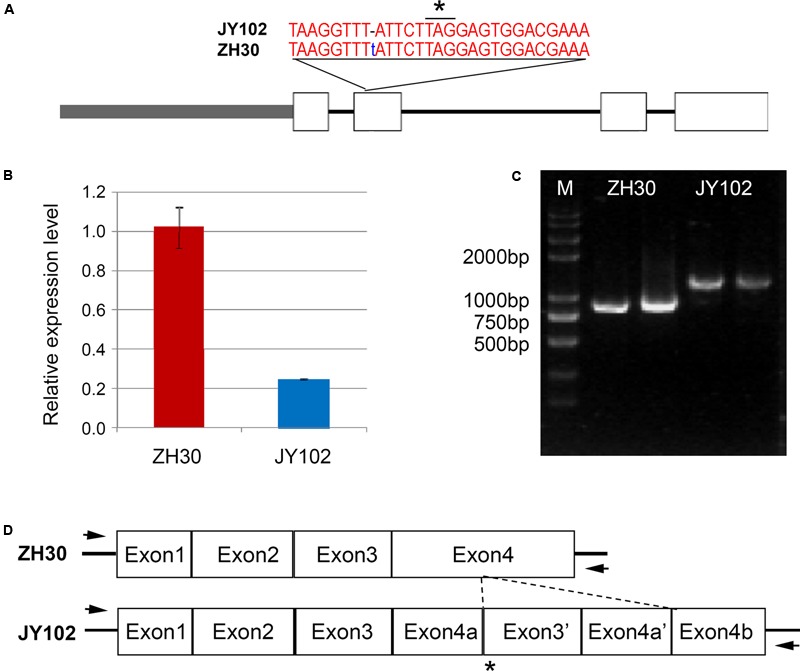
Sequencing confirmation of candidate genes of *qCC1* and *qCC2*. **(A)** Variation of *Glyma.01g214600* in ZH30 and JY102. JY102 has a single-base deletion in the second exon, resulting in a frameshift and premature termination of translation. Star indicated the stop codon. **(B)** Expression of *Glyma.01g214600* in ZH30 and JY102 plants. **(C)** Amplified fragments of *Glyma.11g027400* from ZH30 and JY102. **(D)** Gene structure of *Glyma.11g027400* in ZH30 and JY102. JY102-type *Glyma.11g027400* contains a duplicated copy of exon 3 and exon 4. Star indicated the stop codon and arrows indicated the locations of primers.

The *D1* and *D2* genes involved in the stay-green phenotype of soybean are orthologs of *STAY-GREEN* (*SGR*) genes from *Arabidopsis* (Fang et al., 2014; Nakano et al., 2014). Our fine-mapping region of *qCC1* contained *Glyma.01g214600* (*D1*) and *qCC2* region contained *Glyma.11g027400* (*D2*), indicating the accuracy of our results from mapping by sequencing. According to the SNP and indel set between two parental lines, the deletion of T at position 54,555,967 of chromosome 1 led to a frameshift and premature stop codon in *Glyma.01g214600*, in agreement with the mutation of *D1* in Harosoy near isogenic lines (**Figure 5A**). Primers were designed for sequencing the promoter and genic region of *Glyma.01g214600* and the result revealed that a total of 11 SNPs and a 10-bp indel were identified in the promoter region of *Glyma.01g214600* in JY102. Gene expression analysis also showed that the expression level of *Glyma.01g214600* was significantly lower in JY102 than in ZH30 (**Figure 5B**). For the *qCC2* locus, amplification of the coding sequence of *Glyma.11g027400* revealed that the coding sequence in JY102 was longer than that in ZH30, in consequence of a 322-bp sequence repetition (**Figures 5C,D**), leading to a premature stop codon. This sequence variation is also in agreement with the mutation of *D2* gene identified previously.

## Discussion

Soybean has a paleopolyploid genome that experienced two round of whole genome duplication at about 59 and 13 million years ago. As a result, nearly 75% of annotated soybean genes are present in multiple copies (Schmutz et al., 2010). Compared with diploids, polyploid species usually pose problems for identifying desirable phenotypes in mutant populations, owing to the gene redundancy (Chen et al., 2012). For this reason, only a few mutant libraries have been developed in soybean to date, using fast neutron or ethyl methanesulfonate methods (Bolon et al., 2011; Tsuda et al., 2015; Li et al., 2017), far fewer than mutant resources of *Arabidopsis* and rice (Wang et al., 2013). Although NGS-based BSA approaches have been shown to be efficient in isolating a gene controlling a given mutant phenotype by backcrossing the mutant to the non-mutagenized parental genotype, it is difficult to unequivocally identify the causal mutation due to limited polymorphic markers between mutant and wild-type (Abe et al., 2012; Hartwig et al., 2012; James et al., 2013; Schneeberger, 2014; Huo et al., 2016). Another challenge for crops with large or complex genomes is that some short sequence reads may not be mapped to unique positions in the reference genome and identified nucleotide variations cannot be distinguished from differences among closely related paralogous sequences (Xu et al., 2013). However, different genotypes usually show higher polymorphism than artificial mutants, and massive numbers of high-quality SNPs can be identified by deep sequencing. Thus, populations derived from cultivars harboring useful alleles with natural variants are good resources for NGS-based BSA in crops.

Although reverse-genetic approaches have become increasingly popular in some species in the last few decades, map-based cloning is still the main approach for identifying and isolating candidate genes for many crops. However, the labor-intensive, time-consuming, and costly development of massive marker sets reduces the application and effectiveness of conventional mapping (Lindner et al., 2012). In comparison with model species, only a limited number of genes have now been functionally defined in soybean (Xia et al., 2013). In the present study, we demonstrated the successful application of NGS for simultaneously detecting two genes (*qCC1* and *qCC2*) governing cotyledon color. The major advantage of this method is that it can quickly associate loci with candidate genomic regions, greatly reducing workload and time. Together with the rapid identification of genomic regions, a large number of SNPs and small indels have also been identified between parental lines. Causal mutations of candidate genes may also be identified by detailed analysis of SNPs and indels in candidate genomic regions after fine mapping. In our study, two previously reported stay-green genes isolated by homology-based gene cloning appeared in regions identified by fine mapping, further demonstration of the mapping and polymorphism-identification methodology.

The physical sizes of two mapping regions, of 2.68 Mb in both chromosomes 1 and 11, are still very large even with >50× genome coverage for two bulks, a result consistent with those of other reports in soybean (Campbell et al., 2016; Dobbels et al., 2017). These genomic regions are so large that gene mutations are difficult to identify directly. The limited mapping resolution is primarily due to low recombination rates in mapped intervals and relative small number of bulked samples. Increasing the sequencing coverage and numbers of SNPs cannot increase mapping power unless the number of samples in bulked populations also increases. The other strategy is to backcross the mutant to its parental genotype and the size of a backcrossing population has less influence on the mapping power (James et al., 2013; Schneeberger, 2014). Some successful applications were reported in species with large genomes such as lettuce and wheat (Trick et al., 2012; Huo et al., 2016). In order to fine-map target loci to small regions, subsequently genotyping the progeny of the segregating population is cost-efficient and rapid. The causal mutations of candidate genes can be identified by detailed analysis of SNPs and indels in the relatively small candidate interval regions. The single-nucleotide deletion in the causal gene of *qCC1* (*Glyma.01g214600*), leading to frameshift mutation and loss of function, has also been identified from analysis of the sequencing data. The direct discovery of causal variants of genes in fine-mapping regions dramatically accelerates the speed of candidate gene cloning. However, large-fragment insertions or deletions such as the variant of the *D2* gene still cannot be identified directly from the sequencing data, but must be discovered by sequencing of candidate genes in the mapping regions and further validated.

## Author Contributions

YG and L-JQ conceived and designed the experiments. JS, ZLi, and ZLiu performed the experiments. YG and JS analyzed data, YG, JS, and L-JQ wrote the manuscript. All authors read and approved the manuscript.

## Conflict of Interest Statement

The authors declare that the research was conducted in the absence of any commercial or financial relationships that could be construed as a potential conflict of interest.

## Footnotes

1 http://www.bioinformatics.babraham.ac.uk/projects/fastqc

2 https://phytozome.jgi.doe.gov/pz/portal.html

## Supplementary Material

FIGURE S1 SNP-index of the GC bulk and the YC bulk and Δ(SNP-index) plots generated by next generation sequencing. The *X*-axis shows physical positions on the 20 soybean chromosomes. The *Y*-axis shows the SNP-index estimated for 2 Mb physical intervals with a 10-kb sliding window. Two candidate loci (marked by asterisks) were identified as being associated with cotyledon color in soybean.

TABLE S1 Details of polymorphic markers and primers for PCR amplification.

TABLE S2 Details of SNP-index and annotation for each SNP in the *qCC1* region.

TABLE S3 Detail of SNP-index and annotation for each SNP in the *qCC2* region.

TABLE S4 SNP and indel variations for candidate genes in the fine-mapping region of *qCC1*.

TABLE S5 SNP and indel variations for candidate genes in the fine-mapping region of *qCC2*.
