# Supplementary figures and images for: Next-Generation Sequencing from Bulked-Segregant Analysis Accelerates the Simultaneous Identification of Two Qualitative Genes in Soybean
Source: Front Plant Sci. 2017 May 31;8:919. doi: 10.3389/fpls.2017.00919 (PMC5449466; doi:10.3389/fpls.2017.00919)

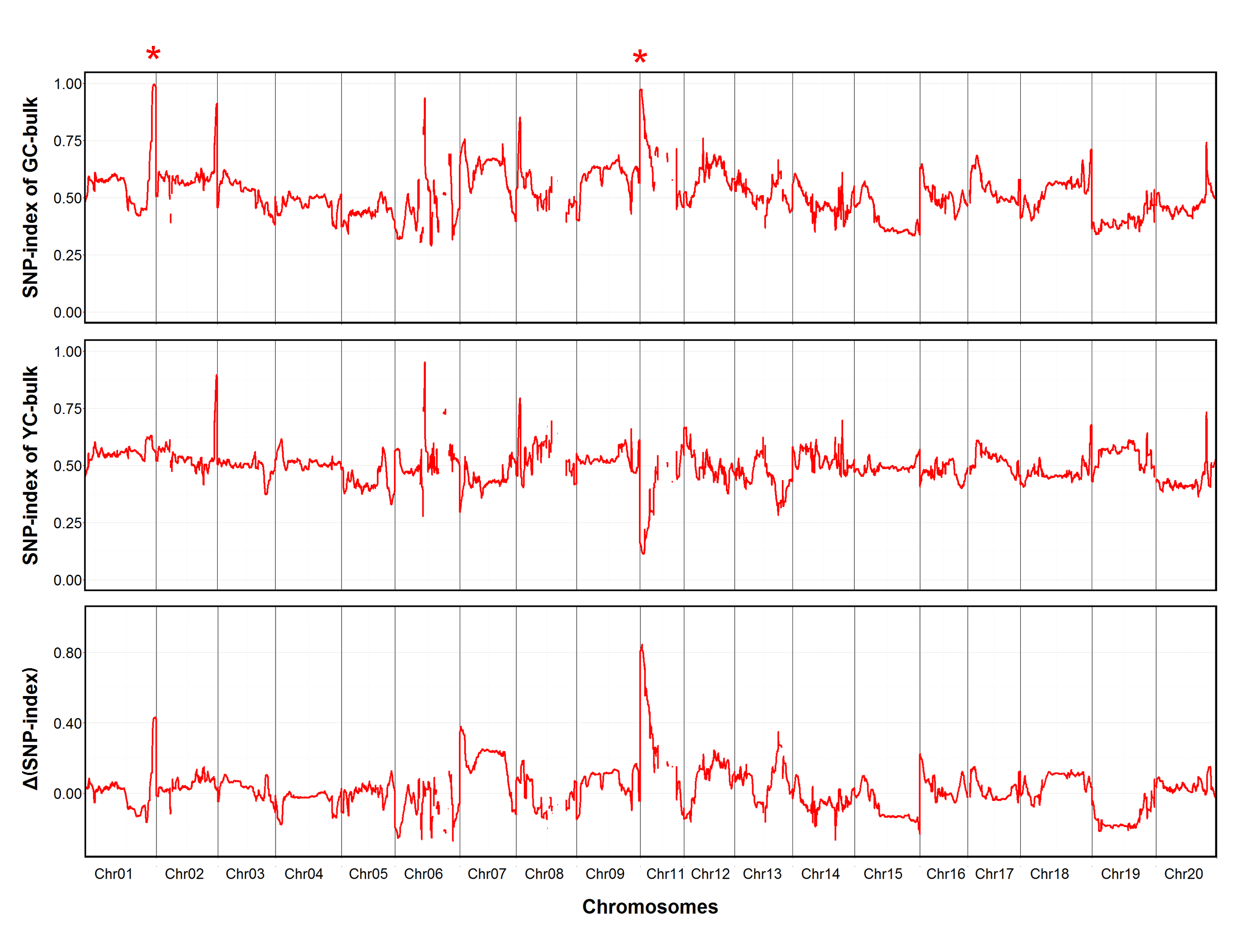

Supplement: FIGURE S1 — SNP-index of the GC bulk and the YC bulk and Δ(SNP-index) plots generated by next generation sequencing. The X-axis shows physical positions on the 20 soybean chromosomes. The Y-axis shows the SNP-index estimated for 2 Mb physical intervals with a 10-kb sliding window. Two candidate loci (marked by asterisks) were identified as being associated with cotyledon color in soybean. [file Image_1.TIF]
